# Neural evidence for image quality perception based on algebraic topology

**DOI:** 10.1371/journal.pone.0261223

**Published:** 2021-12-16

**Authors:** Chang Liu, Dingguo Yu, Xiaoyu Ma, Songyun Xie, Honggang Zhang

**Affiliations:** 1 Institute of Intelligent Media Technology, Communication University of Zhejiang, Hangzhou, Zhejiang, China; 2 College of Media Engineering, Communication University of Zhejiang, Hangzhou, Zhejiang, China; 3 College of Information Science and Electronic Engineering, Zhejiang University, Hangzhou, Zhejiang, China; 4 School of Electronics and Information, Northwestern Polytechnical University, Xi’an, Shaanxi, China; University of Wisconsin-Madison, UNITED STATES

## Abstract

In this paper, the algebraic topological characteristics of brain networks composed of electroencephalogram(EEG) signals induced by different quality images were studied, and on that basis, a neurophysiological image quality assessment approach was proposed. Our approach acquired quality perception-related neural information via integrating the EEG collection with conventional image assessment procedures, and the physiologically meaningful brain responses to different distortion-level images were obtained by topological data analysis. According to the validation experiment results, statistically significant discrepancies of the algebraic topological characteristics of EEG data evoked by a clear image compared to that of an unclear image are observed in several frequency bands, especially in the beta band. Furthermore, the phase transition difference of brain network caused by JPEG compression is more significant, indicating that humans are more sensitive to JPEG compression other than Gaussian blur. In general, the algebraic topological characteristics of EEG signals evoked by distorted images were investigated in this paper, which contributes to the study of neurophysiological assessment of image quality.

## Introduction

In the last decades, with the development of image quality assessment, scientists began to explore the neural mechanism of image quality perception. Neurophysiological approaches are treated as complementary methods to traditional psychophysical ones since quality assessment processes occur inside the media consumer’s brain [1–6]. In the wake of the development of the electroencephalogram (EEG) technique, neurophysiological assessment of image quality becomes more economical and portable [7–16]. Consequently, EEG was taken as an ideal signal to explore the neural responses to image stimuli with different qualities.

Hayashi et al. observed that the value of the power of *α*-EEG was higher in the image with higher quality [7]. Scholler et al. found that quality changes of images evoked an event-related potential called P3 positively correlated with the magnitude of the change [11]. Moreover, since P3 is not directly associated with sensory processing, Steady-State Visual Evoked Potentials (SSVEPs) based paradigm was investigated as a complementary approach by Mueller et al., and the result showed that the adjustment the amplitude of SSVEP had been significantly negatively correlated with Mean Opinion Scores (MOS) [12–14]. In order to elicit the specific EEG signal, a participant is presented a stimulus in the form of corresponding experimental paradigms. For instance, in Mueller’s research [14], the SSVEP signal in the occipital cortex was evoked by the flickering effect of the quality changes at a frame rate of 3 Hz. However, in order to approximate the actual process of human perception of images, images should be presented in a more friendly and natural way. Consequently, in the field of image quality assessment, the display configuration of an image is generally carried out in accordance with a conventional procedure [1, 17] where the evoked EEG signal scarcely has distinctive features. Therefore, when the image is presented according to the conventional image quality assessment paradigm, it is hard to understand the neuropsychological characteristics of quality judgment processes inside the brain sufficiently.

In order to deeply understand the brain’s response to image quality, images need to be presented in a natural way. However, as described above, it is hard to extract the neural characteristics corresponding to perceived quality during the conventional image display procedure. Fortunately, functional network analysis is promising to be a key to solve this dilemma [18–20]. Functional network analysis, which is constructed by quantifying correlations between time series of activity of brain regions, has been rapidly developed since the scientists share the opinion that brain function is determined by the interaction between different neurons and different regions [18, 19, 21, 22]. However, extensive analysis of graphs or networks suffers from a local versus a global problem [23]. Thus, algebraic topology was developed to address the problem. The tools of algebraic topology are uniquely equipped to provide quantitative information about both the local and global properties of a graph, which enables the analysis of the whole brain’s functional patterns without losing the local knowledge of the brain regions. Consequently, Topological Data Analysis (TDA) [24–29] provides a series of new topological and geometric tools to analyze EEG signals. The neural activity distribution emerging in different brain areas will entail different brain patterns, and we expect to find its expression in subtle yet highly significant differences in topological characteristics. Giusti et al. revealed intrinsic geometric structure in neural correlations by clique topology [30]. Using pairwise correlations of neurons in the hippocampus, they detect geometric organization intrinsically from the neural activity without appealing to external stimuli or receptive fields. In subsequent studies, Giusti et al. comprehensively discussed the feasibility of using higher-order algebraic topology tools to understand neural data and presented a basic theoretical framework for brain model computation using algebraic topology for the first time [31]. Furthermore, Santos et al. explored topological phase transitions in functional brain networks, and they considered that a topological invariant, the Euler characteristic, suffices to characterize the sequence of topological phase transitions in the complex network [32]. By applying it to the resting-state fMRI analysis in the Human Connectome Project [33], they show that topological phase transitions occur when the Euler entropy has a singularity, which remarkably coincides with the emergence of multidimensional topological holes in the brain network [34–37]. Meanwhile, Piangerelli et al. design a topological classifier for detecting the emergence of epileptic seizures, in which continuous homomorphic entropy is considered as the classification feature of the EEG signals [38]. According to the result, persistent homology not only provides efficient algorithms for calculating the Betti number of each complex in the families under consideration but also encodes the evolution of nested complex homology groups at different scales. Both the Euler characteristics [39] and the persistent homology [27, 40] are helped to understand the data better and keep stability concerning perturbations or the presence of noise in the EEG signals. Thus, they are likely to reveal the neurological perception procedure of image quality from different perspectives and complement each other to some extent. Consequently, the algebraic topological features of the EEG signal, namely Euler characteristics and the persistent homology, are selected in this paper to analyze the brain responses to images at different distortion levels.

In our study, twenty participants were considered in the conventional image quality assessment procedure, and EEG signals were collected synchronously during the test. The neurophysiological feature of image quality perception was investigated by exploiting Euler characteristics and persistent homology features of the EEG signal. On that basis, how the brain regions are involved in the assessment of perceived quality was interpreted by selecting *Vietoris-Rips* filtration. Finally, the relationship between algebraic topological features of EEG signal and image quality is further discussed in detail.

## Methods

**Fig 1:**
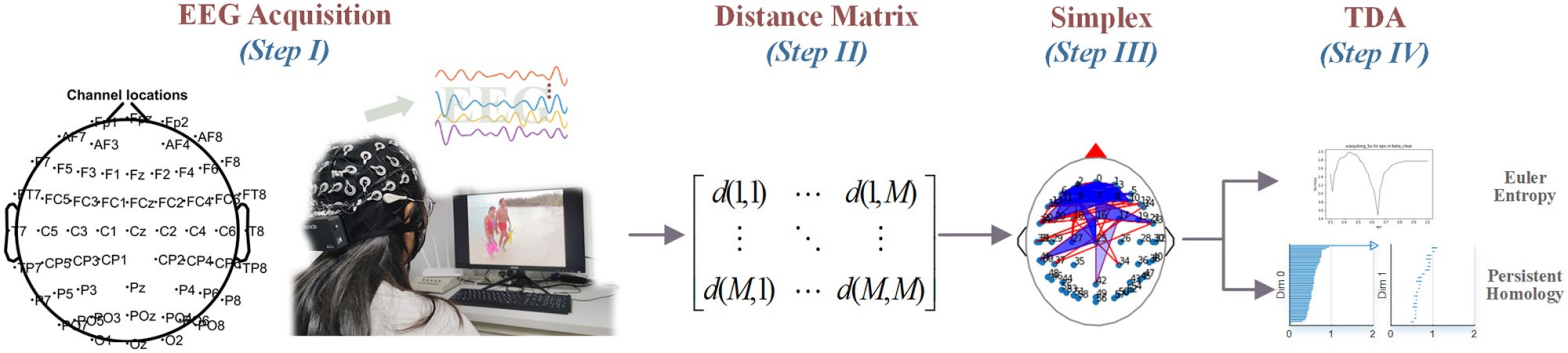
Neurophysiological method for image quality perception analysis. Step I: Collecting the EEG data. Step II: Calculating the distance matrix among electrodes. Step III: Constructing the *Vietoris-Rips* simplex. Step IV: Extracting the algebraic topology characteristics of brain network.

The framework of our proposed neurophysiological method for image quality perception analysis is shown in Fig 1. Collecting the EEG data synchronously by a special cap with electrodes when the subject watches an image with a specific distortion level (Step I). Calculating the distance matrix by correlation among electrodes (Step II) and constructing the *Vietoris-Rips* simplex (Step III). Analyzing the neurophysiological features in the brain stimulated by images across the qualities by TDA (Step IV).

### Stimuli

Forty source images were collected from a subset of the image database in [1], which were freely downloaded from Laboratory for Image & Video Engineering at The University of Texas at Austin. All images had the same mean luminance and were resized to a reasonable size for display on a screen resolution of 1440 × 1080. The LCD monitor is 23.8” (60.5cm). All images were distorted by two distortion types that could occur in real-world applications: Gaussian blur and JPEG compression. Furthermore, in order to maximize the difference, images in two kinds of extreme distortion levels are studied. In Gaussian blur, the standard deviation of the Gaussian kernel of the clear image is 1.5 and that of the unclear image is 16. In JPEG compression, the *Q*-value of the clear image is 70 and that of the unclear image is 8.

### Procedure

**Fig 2:**
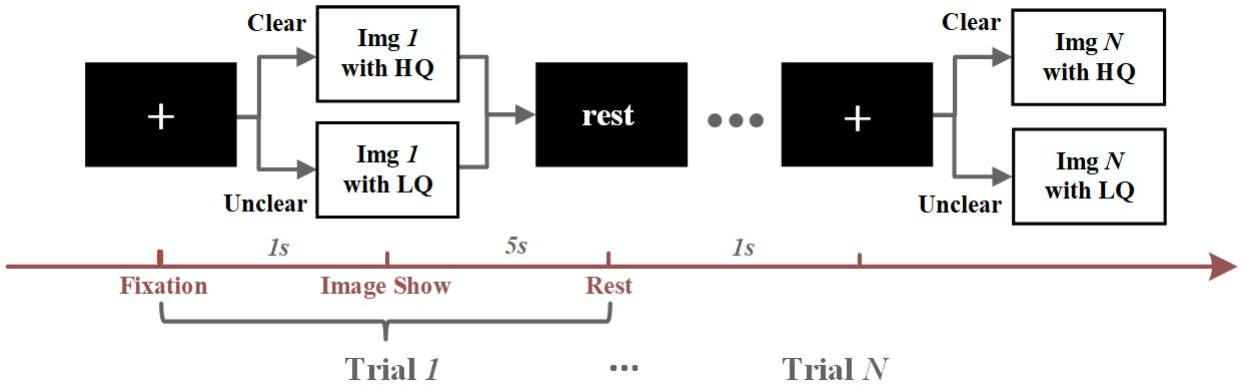
Image quality assessment process. Each trial began with a fixed section lasting 1 second, followed by a randomly presented clear (HQ stands for High Quality while LQ stands for Low Quality) or unclear image lasting 5 seconds, and subjects were required to evaluate image quality. A rest screen was then shown to remind the subjects that they could rest for one second. Then the next experiment began.

In the image quality evaluation test, the subjects were asked to clarify the presented image’s clarity, i.e., clear or not clear. After a general introduction to the experiment and the preparation of the EEG cap, subjects started the test with EEG recording, which is shown in Fig 2. Each distortion type had two sessions, and in each session, subjects had to watch a series of 80 images divided into two classes, i.e., clear image and unclear image. Each trial began with a fixation slice that lasted 1 second, and then a clear or an unclear image appeared randomly for 5 seconds, and subjects needed to assess the quality of the image. A rest slice appeared followed to remind subjects that they could take a break for 1 second. Subsequently, the next trial started to run.

### Subjects and equipment

Data were collected from twenty healthy volunteers (9 males, 11 females; in the age group 19–27) with normal (or corrected to normal) vision, and the subjects had signed the informed consent form. All the subjects were asked to look at the images with a view angle of 20 degrees and rate how clear they were. Test equipment was Neuracle 64 System (Neuracle product; sensor array: 64-channel adult-sized head cap (*M* = 64 in Fig 2); EEG acquisition software: EEG Recorder; amplifier: NSW364; reference electrode: middle of Cz and Pz). The sample rate was 1000 Hz, and the filtering window was 0.3 to 100 Hz.

### Ethical statement

This work (i) identifies the institutional and/or licensing committee that approved the experiments, including any relevant details; (ii) confirms that all experiments were performed in accordance with relevant named guidelines and regulations; and (iii) confirms that informed consent was obtained from all participants and/or their legal guardians. The study is approved by Northwestern Polytechnical University Hospital ETHICS Committee. The individual pictured in ‘Experiment Paradigm.mp4’ has provided written informed consent (as outlined in PLOS consent form) to publish their image alongside the manuscript.

### Topological data analysis for EEG data

Topological data analysis for EEG data is shown in Fig 1 and the following are detailed:

#### EEG acquisition and preprocessing

EEG data are collected by EEG cap and downsampled to 250 Hz. In order to reduce the computation time of topological features, the change of TDA feature of the subjects at different time latencies after the image appeared is investigated first. By comparing the TDA feature of EEG data evoked by a clear verse an unclear image over different latencies after the image is displayed, the EEG of two seconds after displaying the image is selected for the topological data analysis in this paper. A set of filters obtains filtered EEG signals of different wavebands, namely, *δ* band (1∼3 Hz), *θ* band (4∼7 Hz), *α* band (8∼13 Hz), and *β* band (14∼30 Hz)

#### Distance matrix computation

The filtered signal from each electrode in the EEG cap and the distance between electrodes *r* and *t* is calculated by phase-locking value analysis [41, 42] in Euler characteristics analysis:

$$\begin{array}{c} d\left(r,t\right)=\frac{1}{N}|\sum_{n=1}^{N}\text{exp}(j\{\phi^{r}\left(n\right)-\phi^{t}\left(n\right)\})| \end{array}$$ (1)

while in persistent homology analysis, the distance is calculated by standardized Euclidean distance [38]:

$$\begin{array}{c} d\left(r,t\right)=\sqrt{\sum_{k=1}^{N}{\left(\frac{r{|}_{k}}{v_{k}}-\frac{t{|}_{k}}{v_{k}}\right)}^{2}} \end{array}$$ (2)

Here, *j* is the imaginary unit, and *ϕ*^*r*^(*n*) is the *n-th* instantaneous phase of the channel *r* with the length of *N* while *ϕ*^*t*^(*n*) is that of channel *t*. *r*|_*k*_ stands for the *y*-component in (*x*_*k*_, *y*_*k*_) and *v*_*k*_ is the sample standard deviation calculated among all *y*-components at position *k* in channel *r*. Similarly, *t*|_*k*_ stands for the same case of channel *t*.

#### Simplicial complexes construction

**Fig 3:**
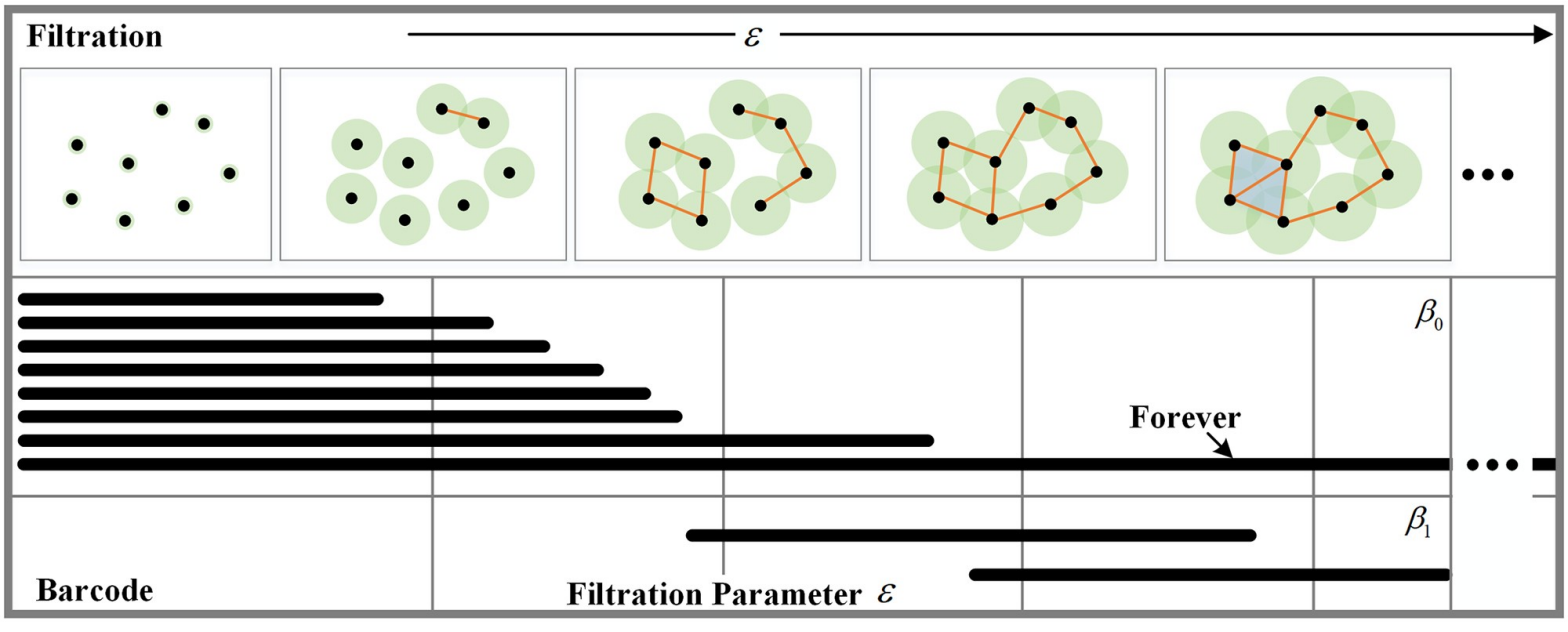
Basic concepts of topological data analysis involved in this paper. *β*_0_ is the number of connected components in *S* while *β*_1_ is the number of holes in *S*. With the increasing of *ε*, a *k*-dimensional hole appears in the simplicial complex.

Simplicial complexes are constructed by *Vietoris-Rips* filtration according to the distance matrix obtained in Step II. A topological structure to an otherwise disconnected set of points is established where a sequence of simplexes is created [23], which is shown in Fig 3. The Betti number of a generic topological space *S* is composed of *β*_0_ and *β*_1_ in this paper. *β*_0_ is the number of connected components in *S* while *β*_1_ is the number of holes in *S*. In fact, according to the findings in the previous study [32], for each Betti curve, it is hard to obtain significant features directly from the Betti curve. Fortunately, further extracting the feature of the Betti curve by Euler entropy or persistent homology entropy will hopefully reveal the perception differences of the subjects stimulated by images in different qualities.

#### Euler characteristics analysis

As shown in Fig 3, each network has an associated topological structure, its simplicial complex, constituted by its nodes (*k* = 0), edges (*k* = 1), triangles (*k* = 2), tetrahedrons (*k* = 3), and higher *k*-dimensional parts. The alternate sum of the numbers of *k*-dimensional simplexes determines the Euler characteristic *χ*. The Euler entropy *S*_*χ*_ of the associated brain network is obtained by the alternate sum of the numbers *Cl*_*k*_ of *k*-dimensional simplexes [32] from *k* = 0 to *k* = *K*, where *K* = 3 is selected as the highest order of simplexes in this paper:

$$\begin{array}{c} \left\{ \begin{array}{c} \langle \chi \rangle =\sum_{k=0}^{K}{\left(-1\right)}^{k}Cl_{k}\left(\varepsilon \right)\\ S_{\chi }=\text{ln}|\langle \chi \rangle | \end{array}\right. \end{array}$$ (3)

Furthermore, a topological phase transition represents a major change in the network topology [23, 32, 34–37], occurring at negative peak with the value of $\varepsilon =\underset{\varepsilon }{\text{arg}\;\text{min}}\left(ln|\chi |\right)$. The phase transition point of Euler entropy occurs at the intersection of Betti curves, and each side of the transition point of Euler entropy represents different topological structures, which means a critical topological change occurs in the brain networks. For instance, before the first transition point, the number of connected components overcomes the number of loops or cycles, and after that point, the situation is reversed. Consequently, the phase transition is selected in this paper to analyze neurological characteristics during the image quality perception. [32].

#### Persistent homology analysis

Persistent homology is an algebraic object that counts the number of *n*-dimensional holes in a topological space, that is, Betti number. During the filtration, time at which a *k*-dimensional hole that appears in the simplicial complex is recorded as *Tstart*, and *Tend* is the time at the *k*-dimensional hole disappears. Accordingly, the *k*-dimensional Betti interval is defined by [*Tstart*, *Tend*], and the persistence barcode is the graphical representation of it [23, 43]. Persistent entropy provides a new entropy measure to extract the feature of topological space by persistence barcode [44–46], which is introduced to measure how “ordered” the structure of the filtering simplex complex is. In this paper, *B* = {(*x*_*i*_, *y*_*i*_)|*i* ∈ *I*} is set to the persistent barcode associated with the filtration of topological space *S*, where *i* is a set of indexes. Moreover Accordingly, the persistent entropy *H* of the filtered simplicial complex is calculated by:

$$\begin{array}{c} H=-\sum_{i\in I}p_{i}\;\text{log}\left(p_{i}\right) \end{array}$$ (4)

where $p_{i}=\frac{y_{i}-x_{i}}{L}$, and *L* = ∑_*i*∈*I*_ (*y*_*i*_ − *x*_*i*_). Moreover, *H* is rescaled and $\hat{H}$ is treated as the persistent homology feature of EEG data in this paper.

$$\begin{array}{c} \hat{H}=\frac{H}{\text{log}\;\ell_{\text{max}}} \end{array}$$ (5)

where *ℓ*_max_ is the maximum interval in the considered persistent barcode.

## Result

### *Vietoris*-*Rips*

**Fig 4:**
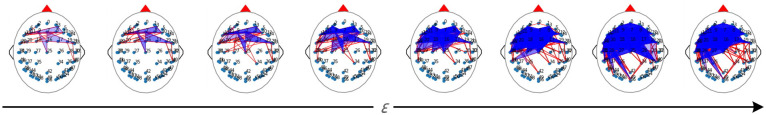
Illustration of the filtration process in a functional brain network where *ε* is from 0.3 to 1.

Topological structures of EEG data evoked by different distortions images are constructed by *Vietoris-Rips* filtration, as shown in Fig 4. A topological structure to an otherwise disconnected set of points is established where a sequence of simplexes is created. In the filtration process, a functional brain network is built for each value of the correlation threshold *ε* ∈ [0, 1] by assigning an edge linking two brain regions if their normalized correlation level is larger than 1-*ε*. As *ε* is enhanced, new edges are gradually attached, thus changing the topology of the brain network, which becomes increasingly denser and harder to analyze. As a consequence, we use Euler characteristic and persistent homology to track these changes in the evolution of both surfaces and networks.

**Fig 5:**
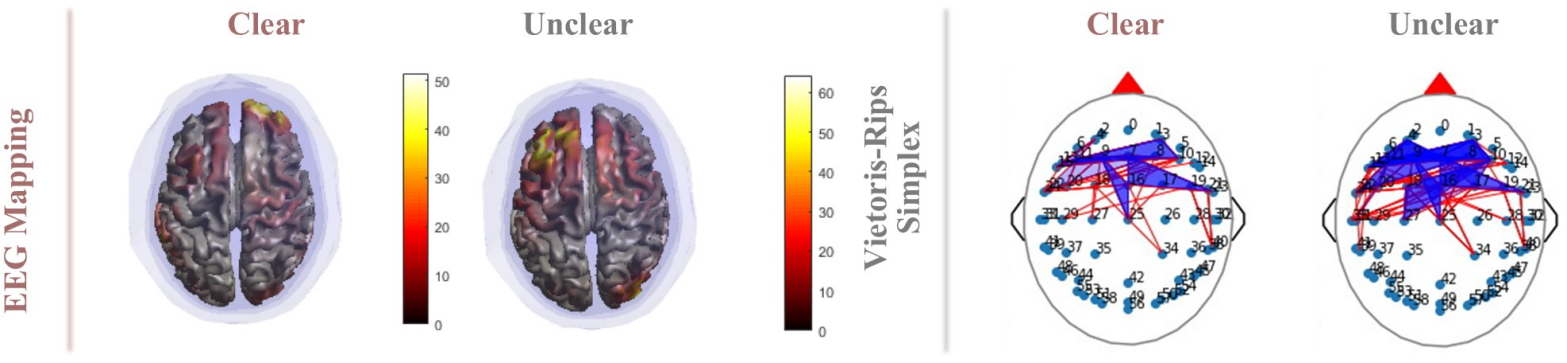
EEG mapping and *Vietoris-Rips* simplex. Red indicates high levels of neuronal activation in EEG mapping. In *Vietoris-Rips* simplex, the red line is the one-dimensional simplex, and the blue parts are the higher-dimensional simplex.

In addition, in order to explore the relationship between different brain regions and image quality perceptions, the EEG mapping result (Model:BEM, template:MNI, source located: MNE) is plotted complimentary in Fig 5, which is the average of the epochs at the same time point (t = 1.36s after image display) under the stimulus of clear and unclear images [47]. Generally speaking, more brain regions are involved when the participant perceives an unclear image compared to a clear one, especially in the frontal lobe. Moreover, the topological structure of EEG elicited by an unclear image is more complicated since the more higher-dimension simplexes are observed. As a consequence, we hypothesize that the blurred images make the task more difficult, leading to more mental activities. These results verify the effectiveness of our approach in describing the complex correlation between EEG signals and image quality perception, and our approach is closer to the actual biological response process.

### Euler characteristics

**Fig 6:**
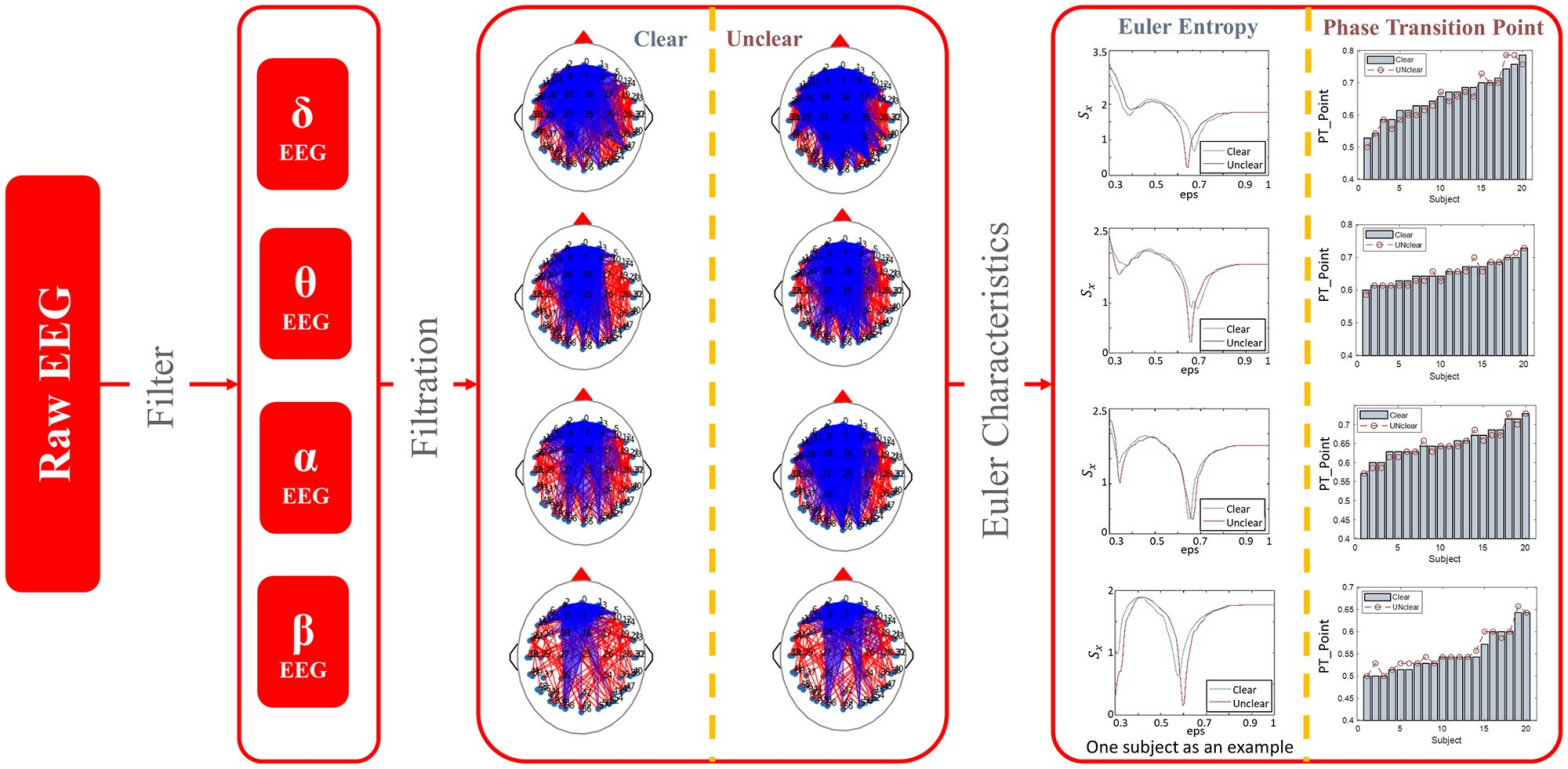
Topological data analysis using Euler characteristics in functional brain networks. The functional networks of EEG data with different frequencies were constructed by algebraic topology analysis. By calculating the variation curve of Euler entropy with parameter *ε* of the network, the corresponding phase transition points are obtained.

Since different frequencies of EEG imply different brain states, the EEG signals of different frequency bands were analyzed respectively [48]. Filtered EEG signals of different wavebands are obtained by a set of filters, namely, *δ* band (1∼3 Hz), *θ* band (4∼7 Hz), *α* band (8∼13 Hz), and *β* band (14∼30 Hz). As shown in Fig 6, filtered EEG signals of different wavebands are obtained by a set of filters. The topological structures of it are constructed by *Vietoris-Rips* filtration, where the phase-locking value (PLV) of EEG data is taking as the normalized correlation coefficient between electrodes. The differences of topological structures of EEG data evoked by different distortions images are consistent across different frequency bands. As mentioned above, the topological structure of EEG elicited by an unclear image is more complex. The Euler entropies of brain networks for different values of *ε* are calculated, and it is a remarkable fact that Euler entropy has a negative peak with the change of *ε*. Since the *ε* value corresponding to the negative peak of Euler entropy is different between the clear and unclear situation, we further calculate the *ε* value when the negative peak of Euler entropy appears, which is taken as a phase transition point in this work. We infer that the value of the control parameter *ε* at which the phase transition occurs is a fingerprint that can be used to differentiate it from others, which is promising to extract the feature of the brain network associated with image quality perception. According to the comparison in different frequency bands among all participants, the phase transition points of Euler entropy differ significantly in the alpha and beta band. Therefore, we focus on analyzing the alpha and beta bands of EEG signals.

**Fig 7:**
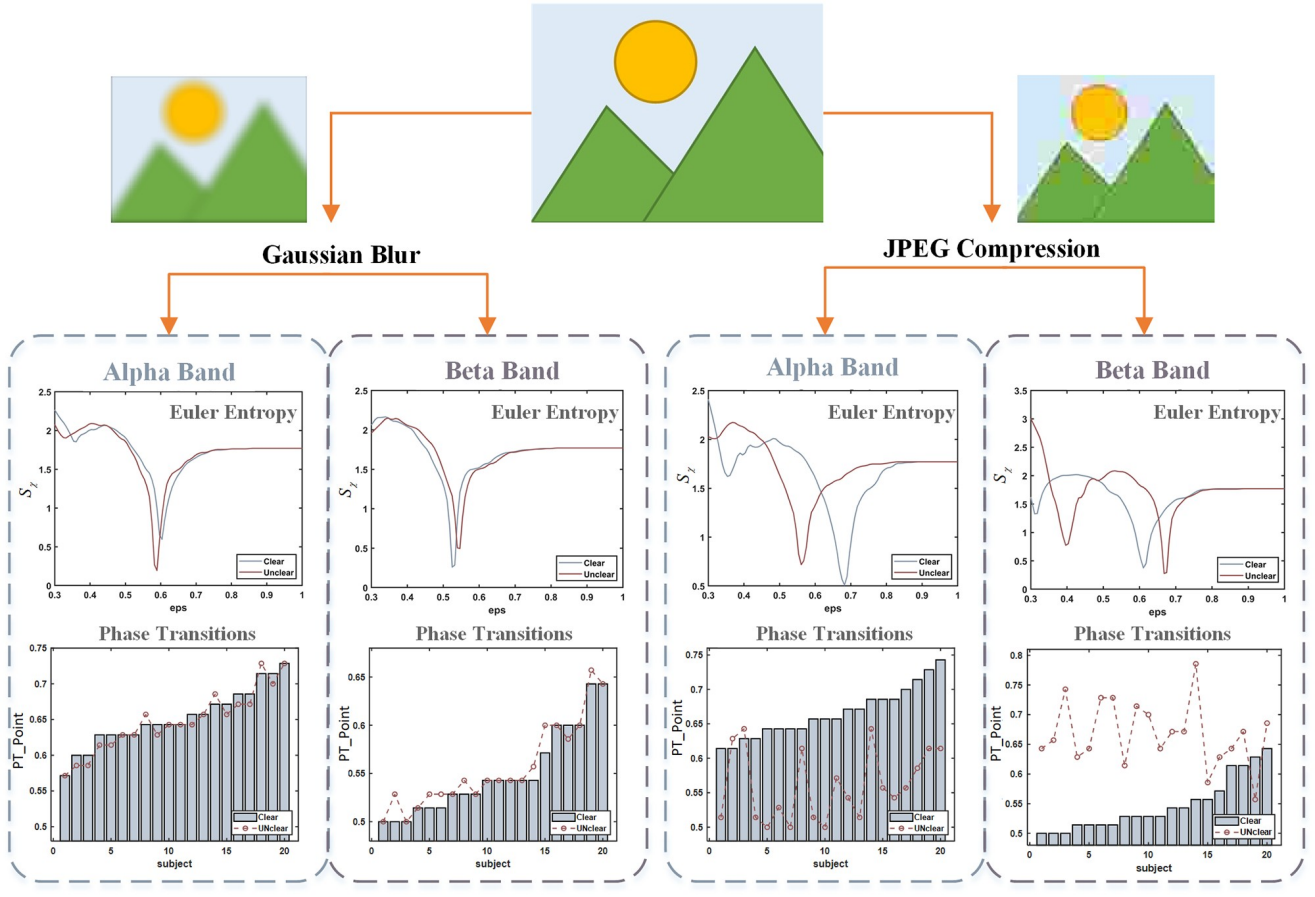
Euler characteristic of brain network constructed by EEG signal in alpha and beta bands evoked by forty source images in clear and unclear deterioration levels. The effect of Gaussian blur on Euler entropy is on the left, while that of JPEG compression is on the right.

Both in Gaussian blur, as well as JPEG compression situations, Euler characteristics of brain network constructed by EEG signal in beta and alpha bands evoked by images in clear and unclear deterioration levels are shown in Fig 7. According to Fig 7, the phase transition point of Euler entropy in the brain network evoked by an unclear image is later than that of the clear image in the beta band. In contrast, the opposite is true in the alpha band, whether Gaussian blur or JPEG compression. Moreover, by comparing the different distortion types, it is evident that the phase transition difference of brain network caused by JPEG compression is pronounced (Paired Samples Test, p<0.05), which indicates that participants may be more sensitive to JPEG compression other than Gaussian blur.

### Persistent homology analysis

**Fig 8:**
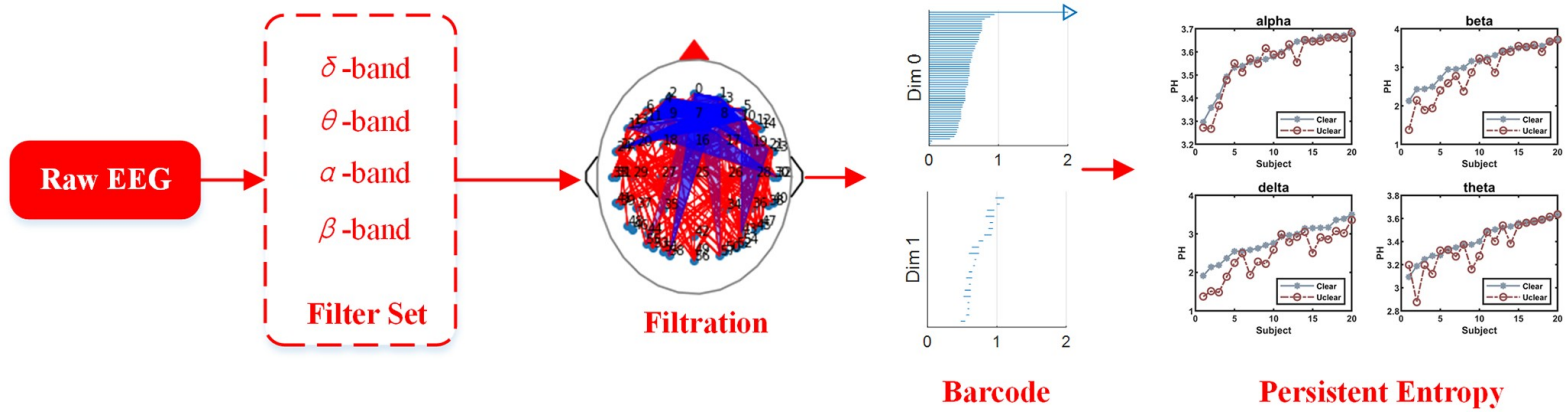
Topological data analysis using persistent homology in functional brain networks. Filtered EEG data by different frequency bands was analyzed by TDA. After filtration, the functional brain network was constructed, and the barcodes were calculated by it. According to the barcodes, the comparison of persistent entropy stimulated by clear and unclear images was shown in different bands.

As shown in Fig 8, similar to Euler characteristic analysis, EEG signal is also analyzed in different frequency bands. After filtration, the functional brain network of EEG in different bands was constructed, and the barcode of it is further calculated by javaplex toolbox [43] to obtain the persistent entropy.

**Fig 9:**
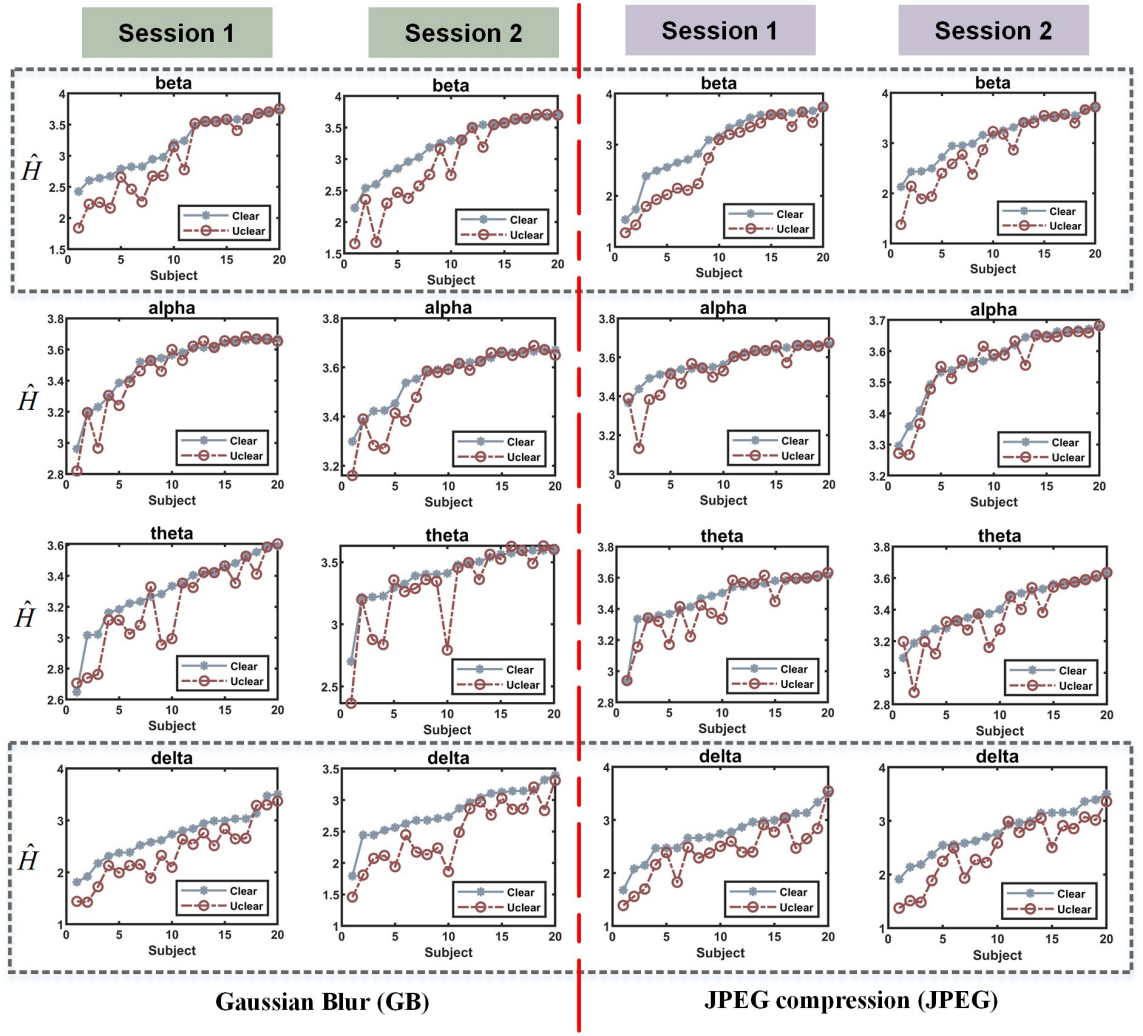
The average value of persistent entropy of EEG signals evoked by forty source images in clear and unclear deterioration levels among different frequency bands. Gaussian blur is on the left, while JPEG compression is on the right. The *y*-axis is the persistent entropy $\hat{H}$, and the *x*-axis is the subject list sort by the PH of the situation from smallest to largest.

Fig 9 shows the average value of persistent entropy of EEG signals evoked by forty source images in clear and unclear deterioration levels. Obviously, the separation of persistent entropy between two brain patterns under different distortions images is clearly depicted in Fig 9, where $\hat{H}$ is plotted. According to Fig 9, the topological structure of EEG evoked by a clear image (solid blue line) has a higher persistent entropy than that of an unclear image (dash red line). It is evident from Fig 9 that there is a strong separation between the two populations, especially in beta and delta bands. Moreover, the persistent entropy of EEG data evoked by clear image and unclear image remains similar differences over the distortion types and sessions.

Furthermore, we perform a paired T-test on persistent entropy of two frequency bands, namely, beta band and delta band. According to the T-test, there are significant differences between the two distortion situations since the p<0.05 both in the beta band as well as delta band.

## Discussion

In this paper, we investigate the neurophysiological processes of image quality perception exploiting EEG. More brain regions, especially in the frontal area, are involved when a subject perceives an unclear image compared to a clear image, which indicates that human perception of image quality might be related to the advanced cognitive processes to some extent. Furthermore, TDA is adopted to extract the physiologically meaningful feature of EEG evoked by different deterioration levels. According to Euler characteristics of brain network constructed by EEG signal, both in Gaussian blur and JPEG compression tests, the phase transition point of Euler entropy in the unclear situation in the beta band is later than that of the clear situation. In contrast, the opposite is true in the alpha band. According to the Gradient Magnitude Similarity Deviation (GMSD) analysis of forty images, the quality gap between GB is larger than JPEG(In GB blur, Clear: 0.9317, Unclear: 0.7380; in JPEG compression, Clear: 0.9413, Unclear: 0.8365;). However, the phase transition difference of brain network caused by JPEG compression is more significant, which indicates that human visual systems are more sensitive to additive noise (such as block-wise effect) compared to the loss of detailed information (such as edge information). Meanwhile, according to the persistent homology analysis, the persistent entropy of EEG data induced by clear images is significantly higher than that of unclear images in the beta and delta bands, which indicates that unclear images activate more orderly brain functional responses. Consequently, phase transition of the brain network is associated with image quality in the beta and alpha band while the persistent entropy is in the beta and delta band, which indicates that entropy analysis and persistent homology analysis are complementary to each other. Furthermore, since both Euler characteristics and persistent entropy in the beta band of EEG signal evoked by the image of different deterioration levels have significant changes, beta bands should be closely related to subjects’ perception procedure of image quality.

Moreover, since the statistically significant changes of different distortion levels were found both in Euler characteristics and persistent homology, the TDA analysis investigated in this paper provides a utility neurophysiological assessment of image quality. Here, we have provided some evidence for such applications by comparing topological features among the images with different qualities. Further studies on this issue will be left for future work.

Besides, since intense brain activities are observed in the frontal lobe, the frontal lobe’s function needs to be further explored. The frontal lobe is the physiological basis of the most elaborate psychological activities, responsible for planning, regulating, controlling people’s psychological activities, etc., playing an essential role in the advanced and purposive behavior of humans. It suggests that human perception of image quality might be related to the advanced cognitive processes to some extent. However, many other explanations can account for this pattern of recruitment. As a consequence, frontal recruitment needs to be further investigated in future work.

Compared to the neurophysiological analysis based on the evoked potential that needs a specific experiment paradigm, this work investigated the brain responses during the conventional image assessment test, which is more similar to the actual human perception of images, and the result ought to be more general.

Since this paper focuses on an extreme case of distortion, future studies may aim at more distortion level analysis. In addition, the correlation between brain response analysis and conventional behavioral methods such as MOS ought to further investigate to deeply understand the perception feature of the brain related to image quality.

## Conclusion

In this paper, we proposed an approach for neurophysiological assessment of image quality from EEG using algebraic topology characteristics of the brain network. Our approach acquired quality perception-related neural information by integrating the EEG collection with conventional image assessment procedures and obtaining physiologically meaningful characteristics of brain responses to different distortion-levels images by topological data analysis. According to the validation experiment results, statistically significant discrepancies of EEG data evoked by a clear image compared to that of an unclear image are observed in several frequency bands, especially in the beta band. The phase transition point of Euler entropy in the unclear situation in the beta band is later than that of the clear situation, while the opposite is true in the alpha band. Meanwhile, the persistent entropy of EEG data induced by clear images is significantly higher than that of unclear images in the beta and delta bands. Since both Euler character and persistent entropy in the beta band of EEG signal evoked by the image of different deterioration levels have significant changes, beta bands should be closely related to subjects’ perception procedure of image quality. Furthermore, the phase transition difference of brain network caused by JPEG compression is more significant, indicating that humans are more sensitive to JPEG compression other than Gaussian blur. In general, the algebraic topological analysis of EEG signals evoked by distorted images is investigated in this paper, and the EEG features related to image quality are obtained, which provides a practical approach for the neurophysiological assessment of image quality.

## Supporting information

S1 Video Animated version of Fig 2. (MP4)
